# Androgen Receptor T878A and L702H Alterations Define Subsets of Prostate Cancer Patients with Distinct Outcomes to Androgen Receptor Pathway Inhibitors

**DOI:** 10.3390/ijms27188199

**Published:** 2026-09-15

**Authors:** Matthew Siskin, Jayati Saha, Emmanuel S. Antonarakis, Alessandro Leal, Samuel Parry, David R. Wise

**Affiliations:** 1 Perlmutter Cancer Center, NYU Langone Health, 160 E 34th Street, New York, NY 10016, USAdavid.wise@nyulangone.org (D.R.W.); 2 Guardant Health, 3100 Hanover St., Palo Alto, CA 94304, USA; jsaha@guardanthealth.com; 3 Masonic Cancer Center, University of Minnesota, 909 Fulton Street SE, Minneapolis, MN 55455, USA; anton401@umn.edu

**Keywords:** prostate cancer, mAPMR, mCRPC, androgen receptor, cfDNA, precision medicine, ARPI

## Abstract

Metastatic androgen pathway modulation-resistant (mAPMR) prostate cancer subtypes harboring *androgen receptor* (*AR*) alterations have distinct AR biology that may impact response to therapy. We hypothesized that patients harboring *AR* T878A would have superior responses to AR pathway inhibitor (ARPI) therapy based on prior preclinical data. We utilized a large genomic-clinical database to identify mAPMR patients with *AR* T878A, *AR* L702H and *AR* amplification and performed a matched assessment of ARPI treatment outcomes using real-world surrogate endpoints for treatment response. Among *AR* T878A patients treated with enzalutamide, time to treatment discontinuation (TTD) was longer relative to patients with *AR* L702H (8 mo (95% CI 4.6–44 mo) vs. 3.5 mo (95% CI 2.3–5.5 mo) log-rank *p* < 0.0001) or *AR* amplification (7.7 mo (95% CI 4.7–15.8 mo) vs. 4.8 mo (95% CI 4–5.1 mo) (log-rank *p* = 0.0034). Among the *AR* T878A patients treated with abiraterone, TTD was not significantly different relative to patients with *AR* L702H, but longer relative to patients with *AR* amplification (7.1 mo (95% CI 5–8.5 mo) vs. 4.2 mo (95% CI 3.5–5.4 mo) (log-rank *p* = 0.037). This study provides hypothesis generating evidence that outcomes for patients with *AR* LBD mutations may differ by mutation subtype. *AR* T878A may be relatively more sensitive to ARPI treatment than *AR* L702H or amplified *AR*.

## 1. Introduction

In the United States, prostate cancer is the most commonly diagnosed non-cutaneous cancer and is the second leading cause of cancer related death in men. In 2026, it is estimated that 333,830 new cases of prostate cancer will be diagnosed and that prostate cancer will result in 36,320 deaths [1]. Among men diagnosed with prostate cancer in the US, approximately 75% of patients present with localized disease while the remaining present with more advanced disease [2]. Patients with advanced disease can typically be managed successfully with endocrine therapy early in their disease course. However, over time, advanced prostate cancer becomes resistant to this therapy and progresses to metastatic androgen pathway modulation-resistant (mAPMR) prostate cancer. The molecular mechanisms underlying this process have been thoroughly summarized elsewhere [3].

While patients with treatment naïve prostate cancer rarely have *androgen receptor* (*AR*) alterations, a subset of patients with mAPMR prostate cancer have alterations in the *AR* genomic locus, including *AR* amplification and *AR* mutations [4]. Multiple studies have linked these alterations to the development of androgen pathway modulation-resistant disease [3,5]. *AR* amplification (*AR* Amp) is seen in up to 52–80% of patients with mAPMR prostate cancer, while *AR* alterations are found in approximately 20% of patients [4,6,7]. *AR* alterations occur more commonly in patients who have been exposed to second generation androgen receptor pathway inhibitors (ARPIs). Among patients with *AR* alterations, 66% have an isolated alteration while the remainder have multiple co-occurring *AR* mutations. The most commonly encountered *AR* alterations are *AR* L702H and *AR* T878A. Rates of *AR* L702H and *AR* T878A increase with abiraterone treatment. In contrast rates of *AR* L702H increase with enzalutamide treatment but *AR* T878A rates are not affected by enzalutamide exposure [6].

Preclinical studies have demonstrated that *AR* amplification can occur via amplification of the *AR* gene or via amplification of an upstream enhancer [8]. In vitro models of *AR* amplified prostate cancer cell lines have shown decreased sensitivity to AR targeting therapies such as enzalutamide [9]. In a case series of 40 patients with metastatic disease treated with AR targeting therapy, *AR* amplification as detected by a cfDNA based assay was associated with a significantly worse six-month progression free survival (PFS) (30 vs. 71%) and overall survival (OS) (59 vs. 100%) than patients without *AR* amplification [10]. A variety of other studies have similarly shown that *AR* amplification is associated with a poor response to hormonal therapy [11].

The androgen receptor consists of an amino-terminal domain, DNA binding domain, hinge region and carboxy terminal ligand binding domain (LBD). While mutations in various parts of the *AR* gene can impact AR function in a variety of ways, missense mutations in the ligand binding domain can impact prostate cancer disease biology by altering AR ligand binding specificity which allows for AR activation by non-classical ligands and by producing a paradoxical AR activation in the presence antiandrogens [12]. Prior work on the structural impact of *AR* T878A demonstrated that the mutation results in 0.8 Å root mean square deviation relative to the wild-type structure, leaving the overall structure of the AR largely unchanged. Despite this, the mutation results in a change in the binding of DHT to the AR LBD resulting in additional space at position 17 of DHT [13]. The altered binding pocket of the T878A LBD contributes to this AR variants increased sensitivity to alternative ligands such as estradiol and progesterone. *AR* L702H, in contrast, results in a unique network of hydrogen bonds, between histidine and the steroidal 17a hydroxyl group and the serine at position 779 which do not exist in wild-type AR. These changes allow the AR to be uniquely stimulated by steroids with 17-hydroxy groups such as cortisol transforming the AR into a glucocorticoid activated receptor [14]. Importantly the clinical significance of multiple co-occurring *AR* alterations may differ in unique ways from isolated ligand binding domain point mutations. Additionally, in vitro models have demonstrated that *AR* alterations can impact the activity of anti-androgens. In vitro, *AR* T878A remains responsive to enzalutamide but the mutation may negatively impact the binding kinetics of enzalutamide and may convert enzalutamide into a partial AR agonist at high concentrations which is further pronounced with a concurrent *AR* H875Y alteration [15]. This change in enzalutamide pharmacodynamics is not observed with *AR* L702H alterations [16]. In terms of the prognostic implications, prior work has suggested that patients with *AR* LBD mutations in real-world cohorts have a shorter median PFS and OS (50.1 vs. 60.7 mo) relative to patients with wild-type AR highlighting that these patients represent a high-risk cohort that may benefit from targeted management strategies [6,17,18].

Based on the above literature we hypothesized that patients harboring AR T878A may have superior clinical responses to ARPI therapy relative to patients with AR L702H or AR amplification. To test this hypothesis, this study utilized a large genomic-clinical database to identify mAPMR patients with these specific *AR* alterations and performed a matched assessment of ARPI outcomes using real-world surrogate endpoints for treatment response.

## 2. Results

**Figure 1:**
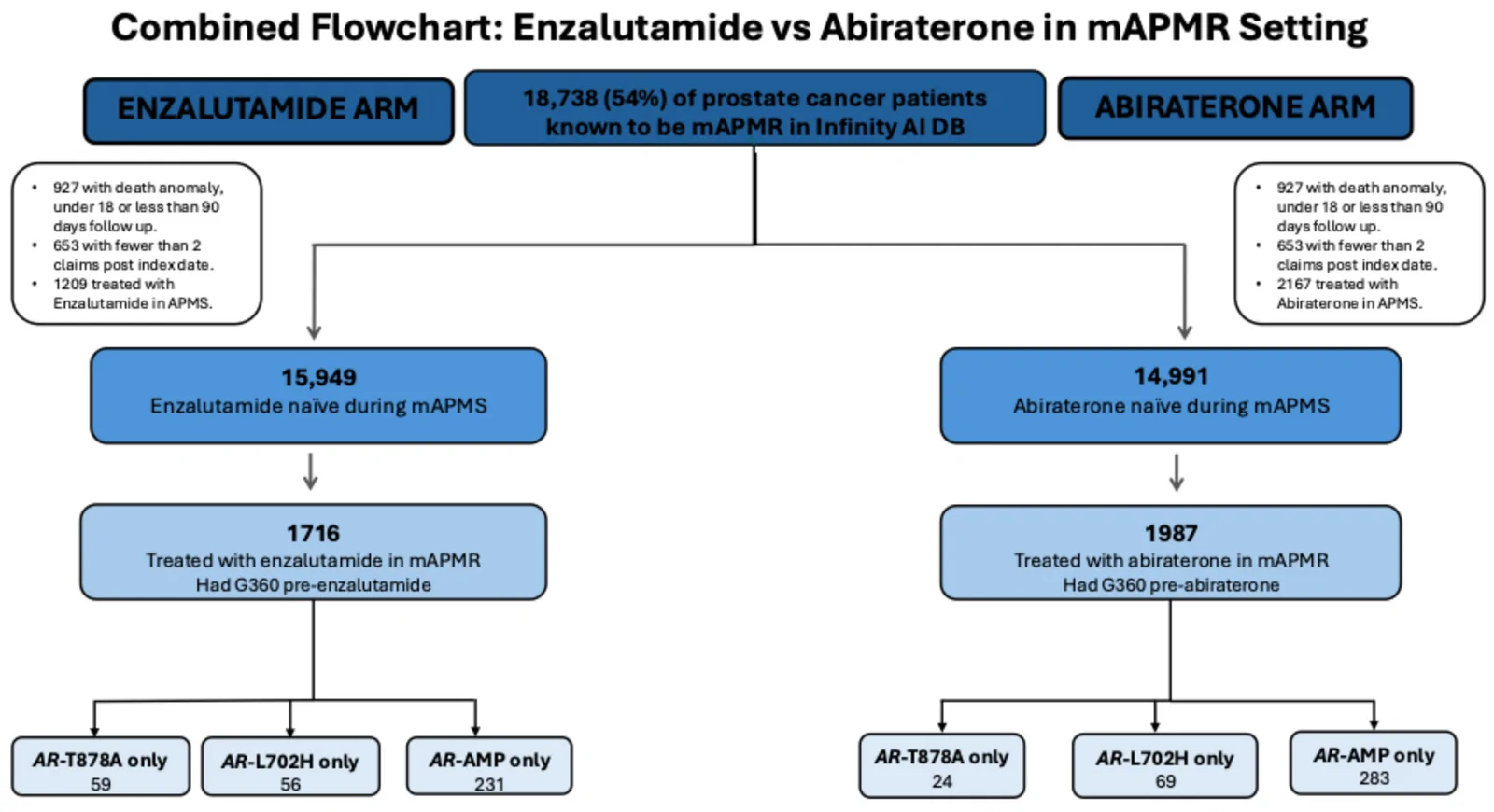
Consort Diagram. mAPMR, metastatic androgen pathway modulation-resistant, mAPMS, metastatic androgen pathway sensitive, AR, Androgen Receptor, *AR*-AMP, Androgen Receptor Amplification.

As of the InfinityAI Data Library database query, there were 34,590 patients with prostate cancer present in the database, of whom 18,738 had mAPMR prostate cancer. In total, 927 had fewer than 90 days of follow up, a death anomaly or were under 18, and 653 had fewer than two claims post index date leaving 17,158 patients potentially eligible. 1209 patients were exposed to enzalutamide in the Androgen Pathway Modulation-Sensitive (APMS) setting, while 2167 were exposed to abiraterone in the APMS setting. Of the remaining patients, 1716 patients were treated with enzalutamide in the mAPMR setting and had a pre-enzalutamide liquid biopsy available and 1987 analogous patients were available in the database who were treated with abiraterone (Figure 1).

Among the potentially eligible patients treated with enzalutamide, 59 (3.4%) had an isolated *AR* T878A alteration, 56 (3.3%) had an isolated L702H alteration and 231 (13.5%) had *AR* amplification. Among the potentially eligible patients treated with abiraterone 24 (1.2%) had an isolated *AR* T878A alteration, 69 (3.5%) had an isolated L702H alteration and 283 (14.2%) had *AR* amplification.

Following matching for age, race, ethnicity, geographic location, comorbidity index and ARPI line of therapy, four cohorts were retrospectively utilized to compare outcomes to ARPI therapy. The characteristics of the four matched cohorts are detailed in Table 1.

**Figure 2:**
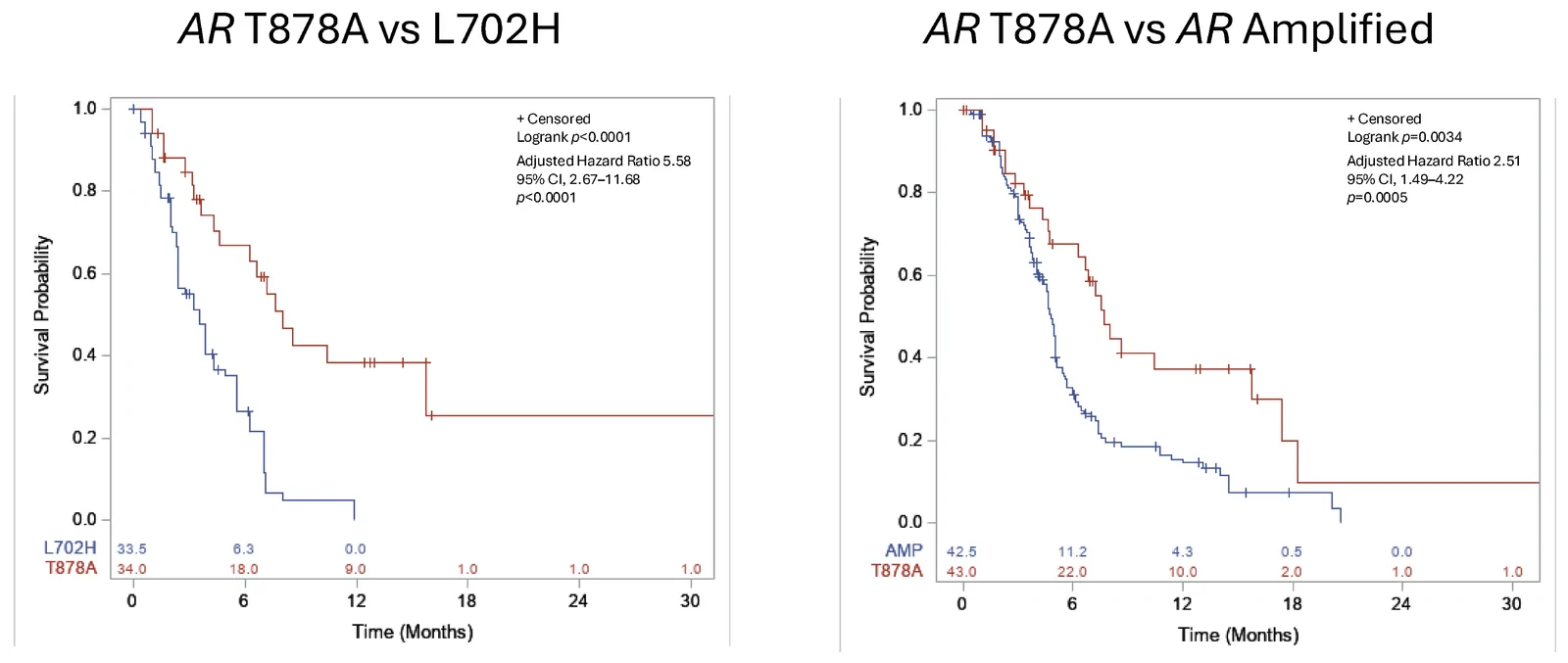
Enzalutamide time to treatment discontinuation (TTD) among patients with *Androgen receptor (AR)* T878A vs. *AR* L702H and *AR* amplification. **Left panel**: Kaplan–Meier (KM) Enzalutamide TTD for patients with *AR* T878A vs. *AR* L702H. **Right panel**: KM Enzalutamide TTD for patients with *AR* T878A vs. *AR* amp. Displayed curves were restricted to 30 mo follow up due to small sample size beyond this timepoint.

**Figure A1:**
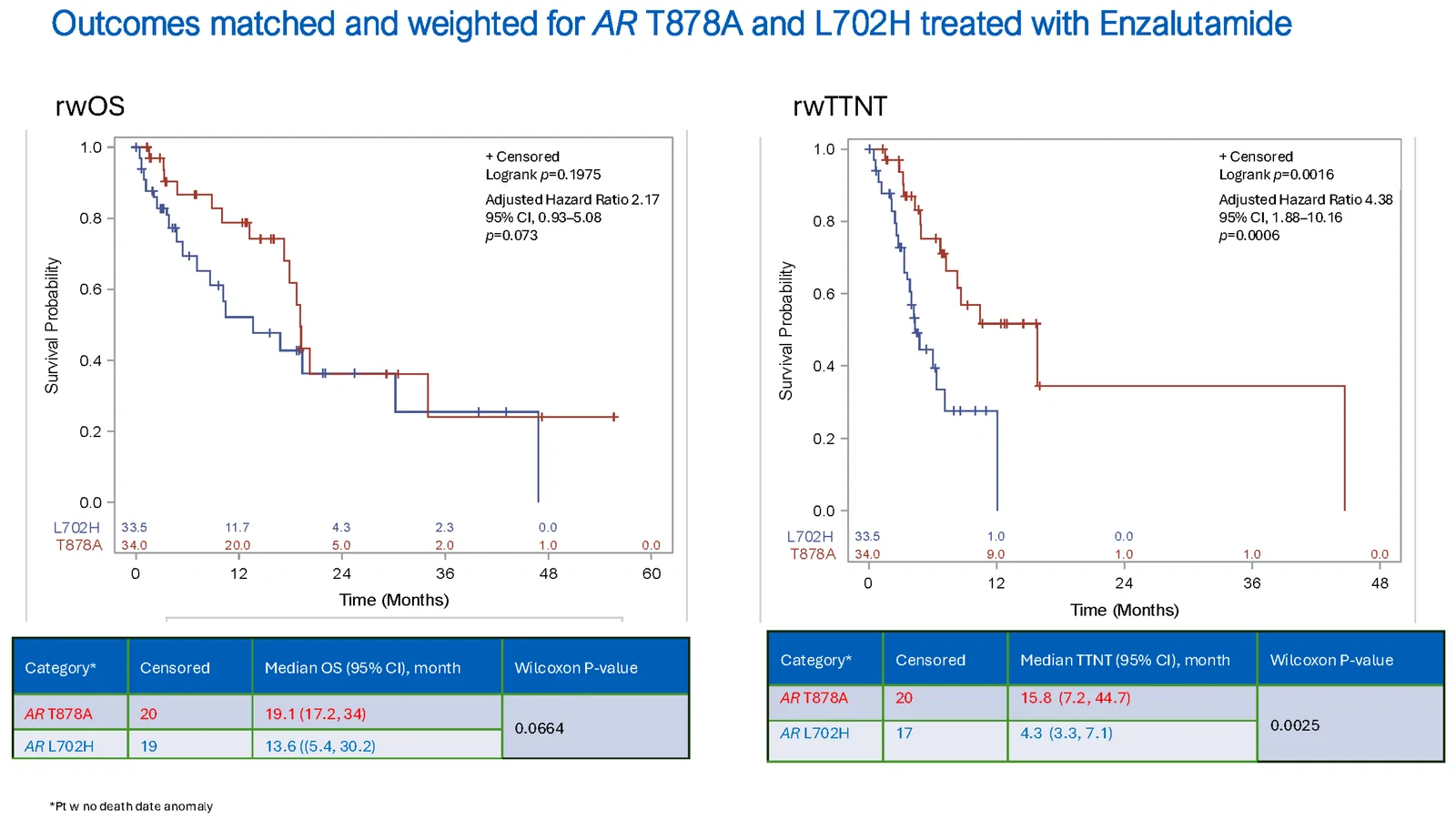
TTNT and OS Cohort A: *AR* T878A vs. *AR* L702H treated with Enza. Left panel depicts KM real-world OS (rwOS). Right panel depicts KM real-world time to next treatment (rwTTNT). Bottom table depicts median OS with 95% confidence intervals and Wilcoxon *p*-value.

**Figure A2:**
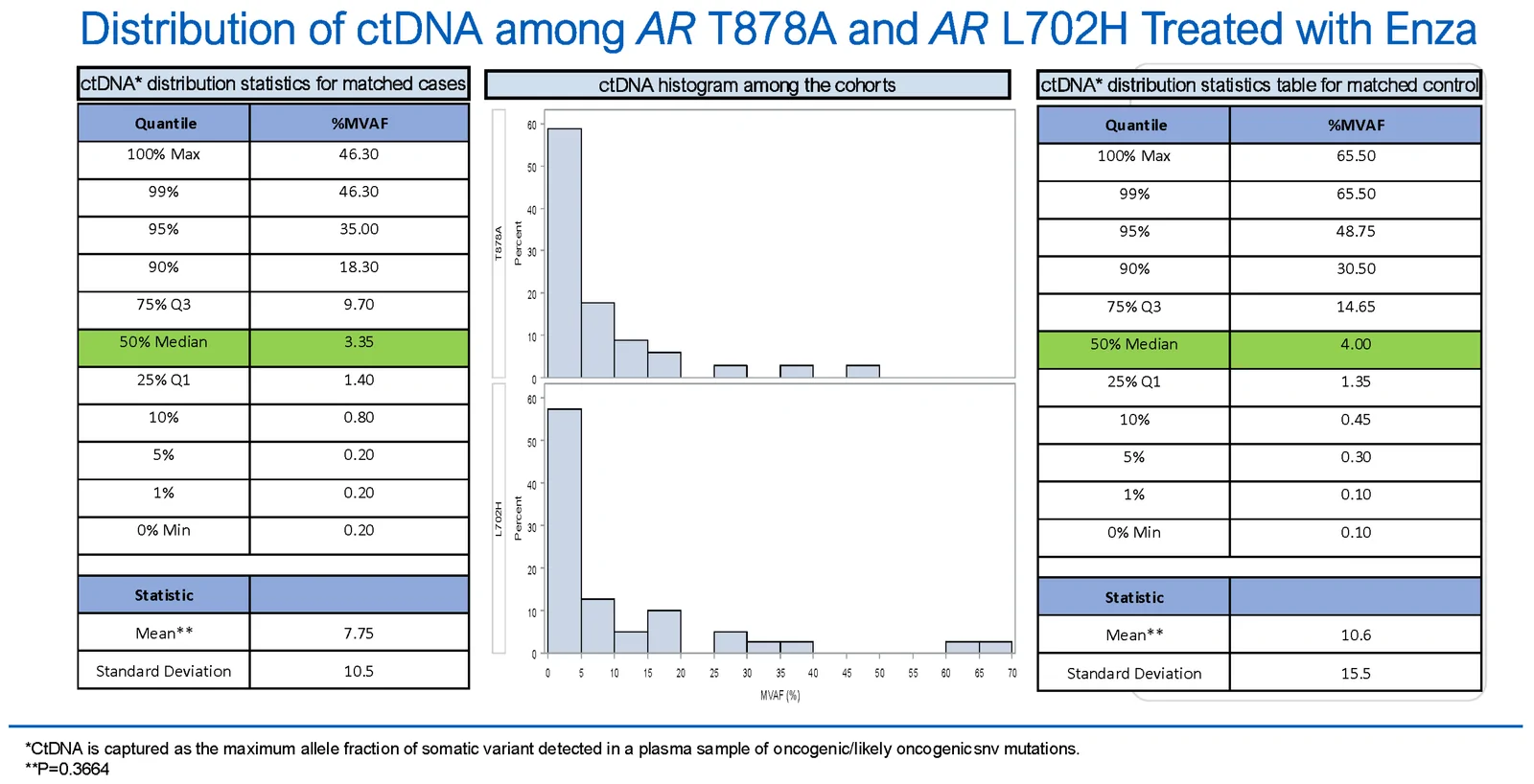
Histogram depicting the distribution of the maximum variant allele fraction detected by cell free DNA among patients in Cohort A with *AR* T878A vs. *AR* L702H treated with Enzalutamide.

In total, 68 patients were included in Cohort A, which assessed how outcomes to enzalutamide therapy among patients with *AR* T878A alterations compared to outcomes among patients with *AR* L702H. All outcomes are presented as *AR* T878A vs. L702H. Adjusted Hazard Ratios use *AR* T878A as the reference group. The median time to treatment discontinuation (TTD) was 8 mo (95% CI 4.6–44 mo) vs. 3.5 mo (95% CI 2.3–5.5 mo) (log-rank *p* < 0.0001, aHR 5.58 95% CI 2.67–11.68 *p* < 0.0001) (Figure 2). The median overall survival (OS) was 19.1 mo (95% CI 17.2–34 mo) vs. 13.6 mo (95% CI 5.4–30.2 mo) (log-rank *p* = 0.1975, aHR 2.17 95% CI 0.93–5.08 *p* = 0.073). The median time to next treatment (TTNT) was 15.8 mo (95% CI 7.2–44.7 mo) vs. 4.3 mo (95% CI 3.3–7.1 mo) (log-rank *p* = 0.0016, aHR 4.38 95% CI 1.88–10.16 *p* = 0.0006). The mean maximum variant allele fraction (MVAF) was 7.75% (95% CI 4.1–11.39%) vs. 10.6% (95% CI 5.65–15.59%) (*p* = 0.3664) (Appendix A Figure A1 and Figure A2).

**Figure A3:**
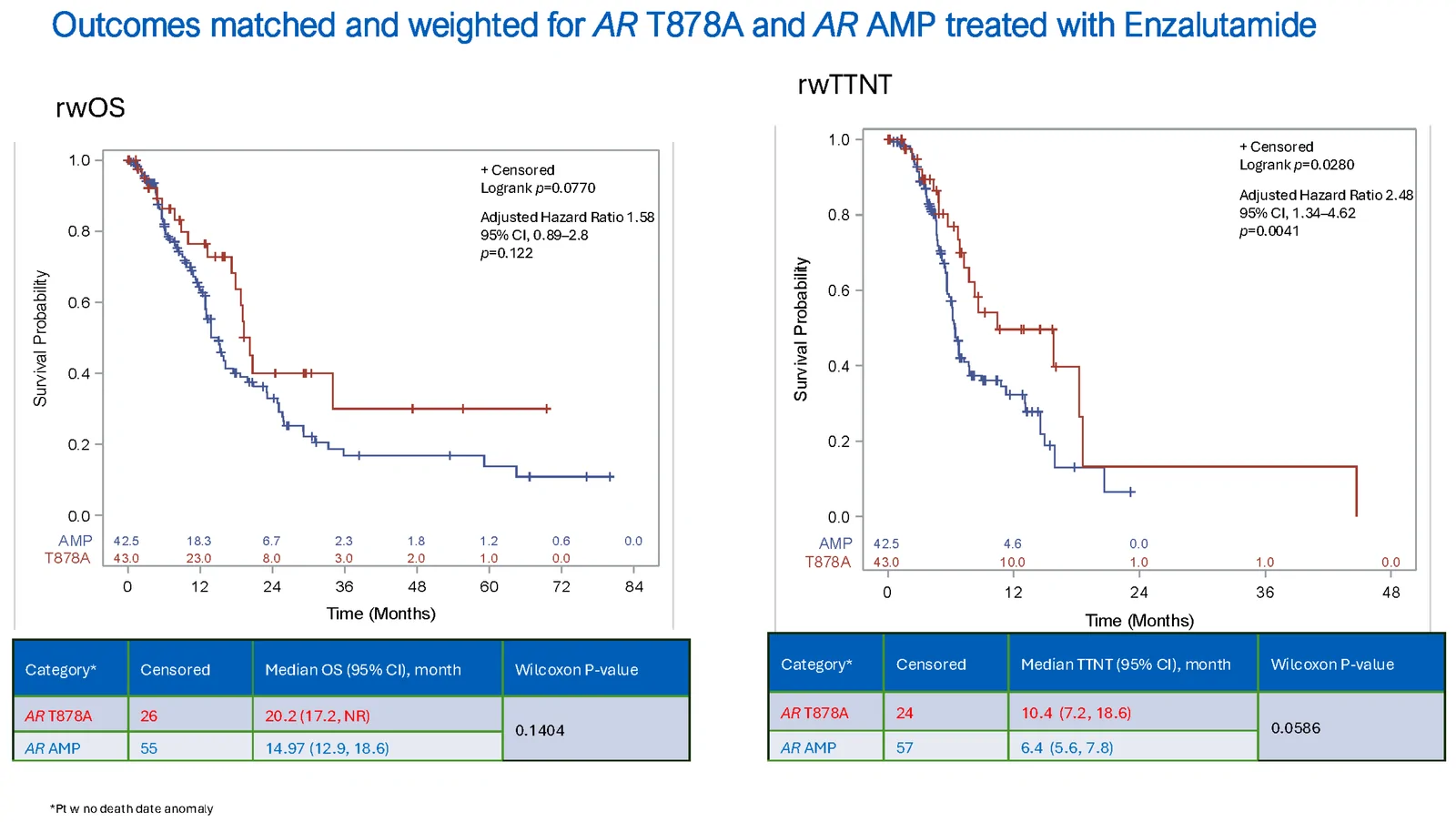
TTNT and OS Cohort B: *AR* T878A vs. *AR* AMP treated with Enza: Left panel depicts KM real-world OS (rwOS). Right panel depicts KM real-world time to next treatment (rwTTNT). Bottom table depicts median OS with 95% confidence intervals and Wilcoxon *p*-value.

**Figure A4:**
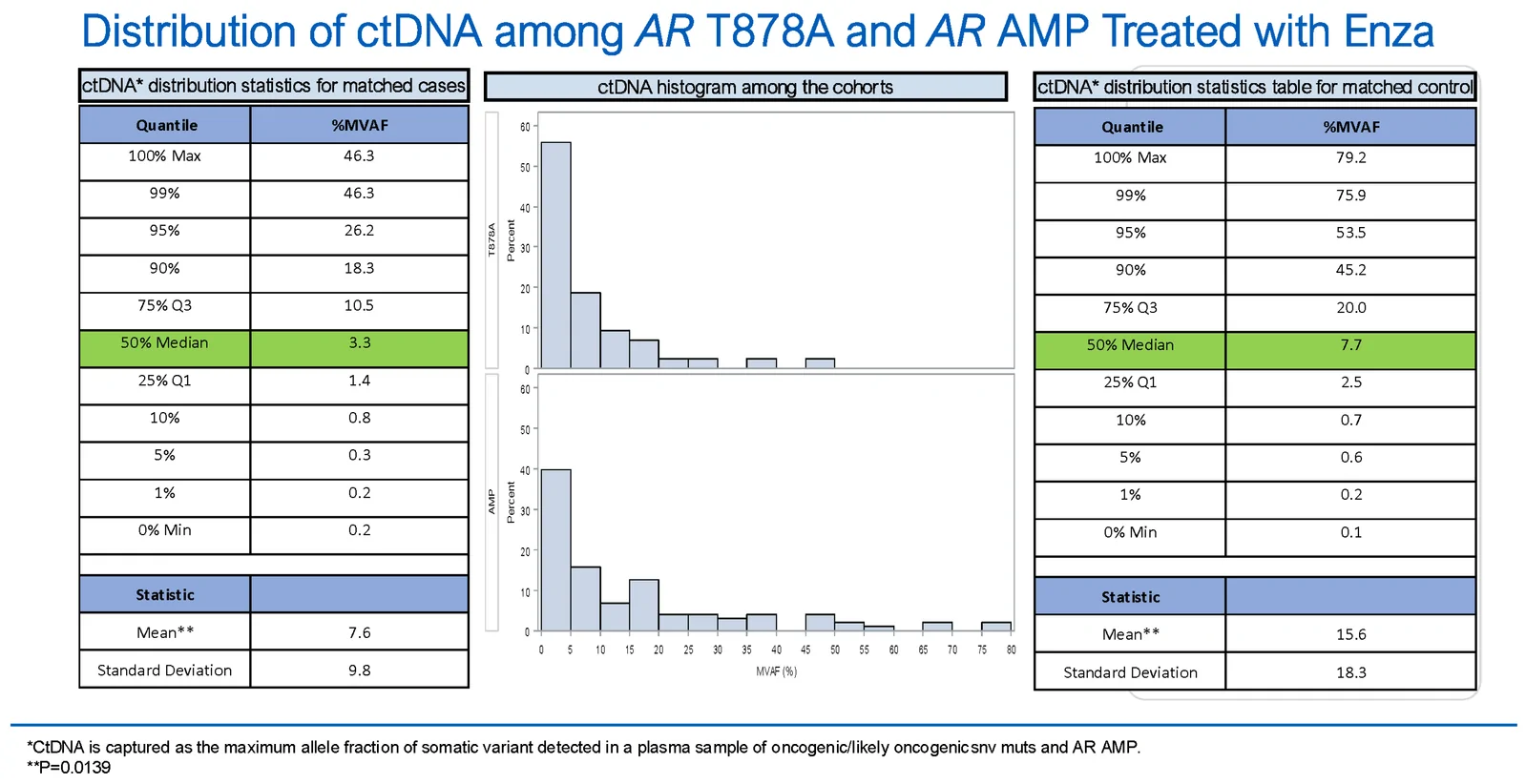
Histogram depicting the distribution of the maximum variant allele fraction detected by cell free DNA among patients in Cohort B with *AR* T878A vs. *AR* AMP treated with Enzalutamide.

In total, 86 patients were included in Cohort B, which assessed how outcomes to enzalutamide therapy among patients with *AR* T878A alterations compared to outcomes among patients with *AR* amplification. All outcomes are presented as *AR* T878A vs. *AR* Amp. The median TTD was 7.7 mo (95% CI 4.7–15.8 mo) vs. 4.8 mo (95% CI 4–5.1 mo) (log-rank *p* = 0.0034, aHR 2.51 95% CI 1.49–4.22 *p* = 0.0005) (Figure 2). The median OS was 20.2 mo (95% CI 17.2 mo-not reached) vs. 14.97 mo (95% CI 12.9–18.6 mo) (log-rank *p* = 0.077, aHR 1.58 95% CI 0.89–2.8 *p* = 0.122). The median TTNT was 10.4 mo (95% CI 7.2–18.6 mo) vs. 6.4 mo (95% CI 5.6–7.8 mo) (log-rank *p* = 0.028, aHR 2.48 95% CI 1.34–4.62 *p* = 0.0041). The mean MVAF was 7.6% (95% CI 4.03–11.12%) vs. 15.6% (95% CI 12.61–18.36%) (*p* = 0.0139) (Appendix A Figure A3 and Figure A4).

**Figure 3:**
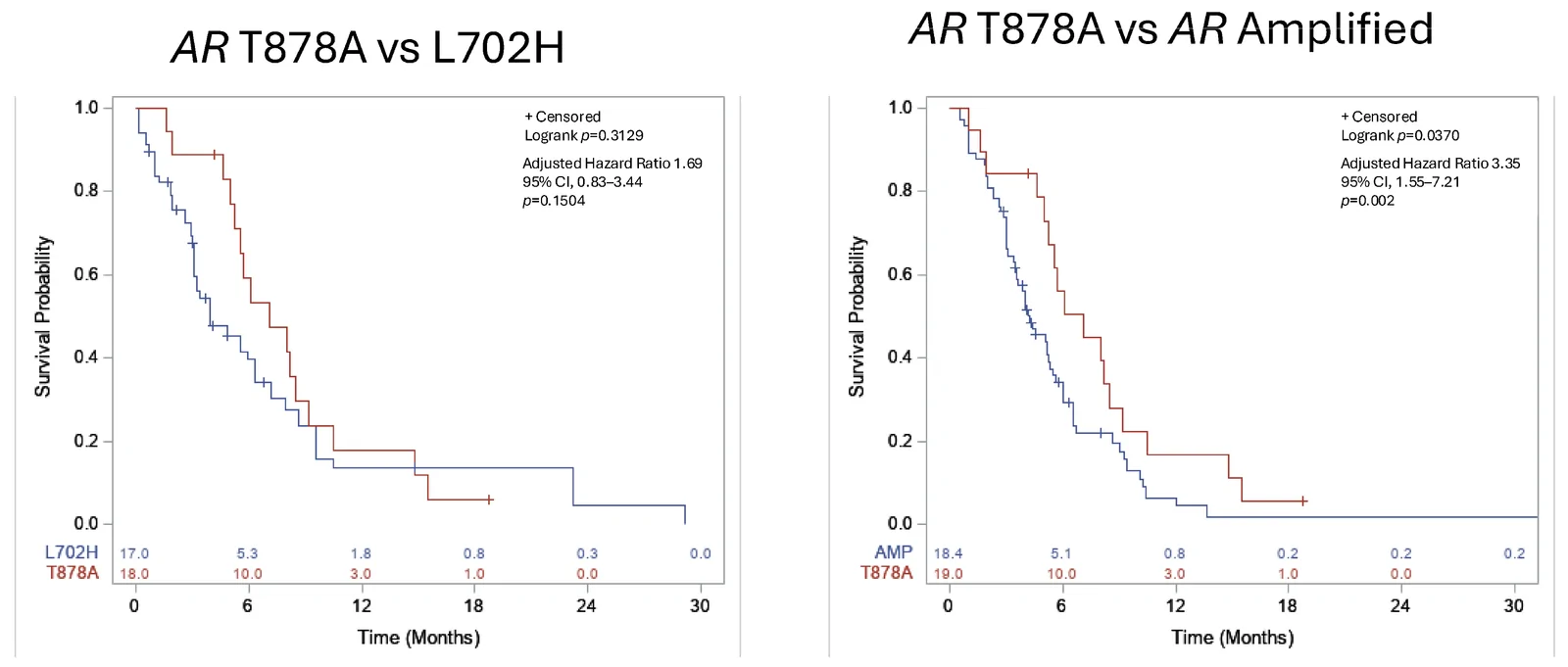
Abiraterone time to treatment discontinuation (TTD) among patients with *Androgen receptor (AR)* T878A vs. *AR* L702H and *AR* amplification. **Left panel**: Kaplan–Meier (KM) Abiraterone TTD for patients with *AR* T878A vs. *AR* L702H. **Right panel**: KM Abiraterone TTD for patients with *AR* T878A vs. *AR* amp. Displayed curves were restricted to 30 mo follow up due to small sample size beyond this timepoint.

**Figure A5:**
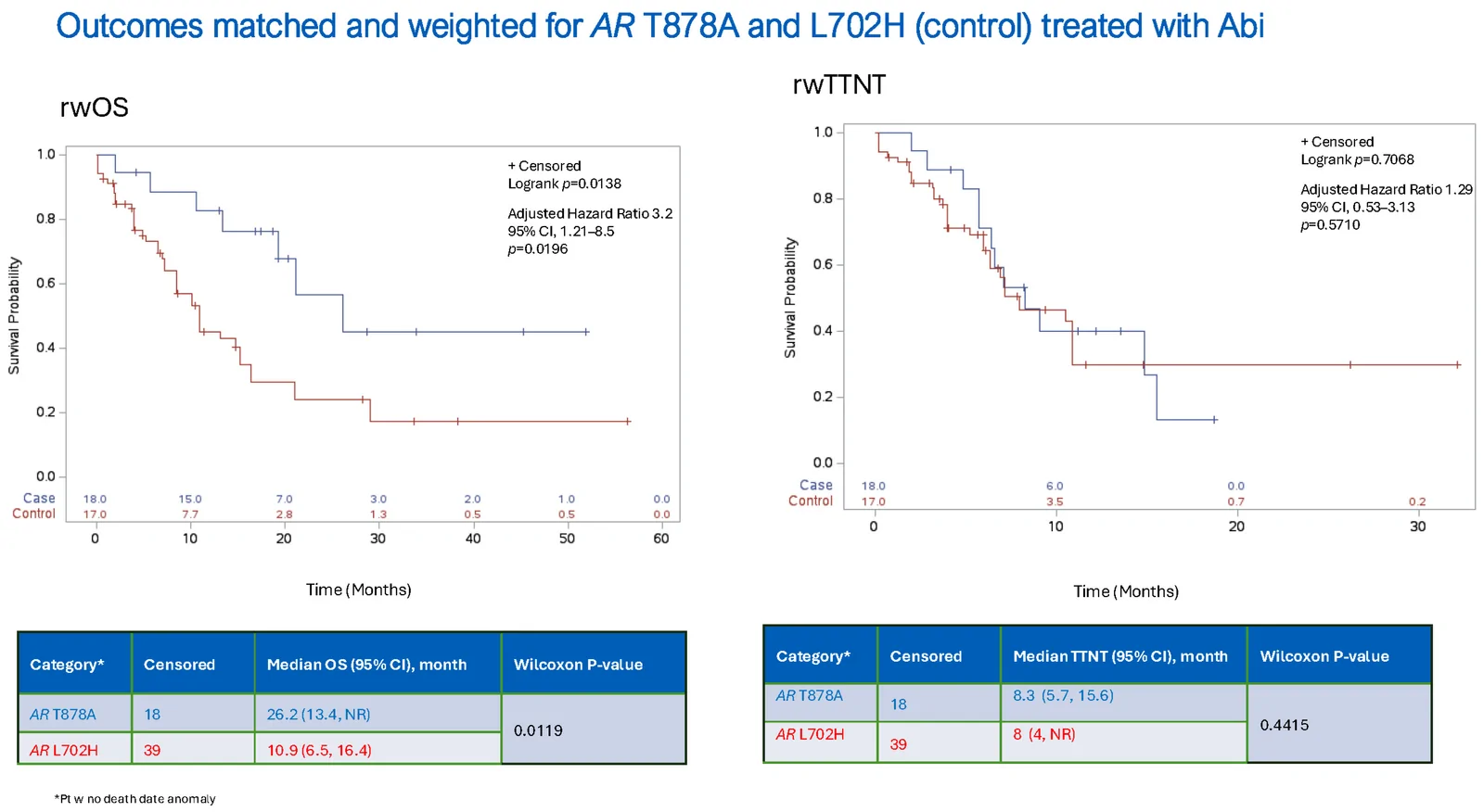
TTNT and OS Cohort C: *AR* T878A vs. *AR* L702H treated with Abiraterone: Left panel depicts KM real-world OS (rwOS). Right panel depicts KM real-world time to next treatment (rwTTNT). Bottom table depicts median OS with 95% confidence intervals and Wilcoxon *p*-value.

**Figure A6:**
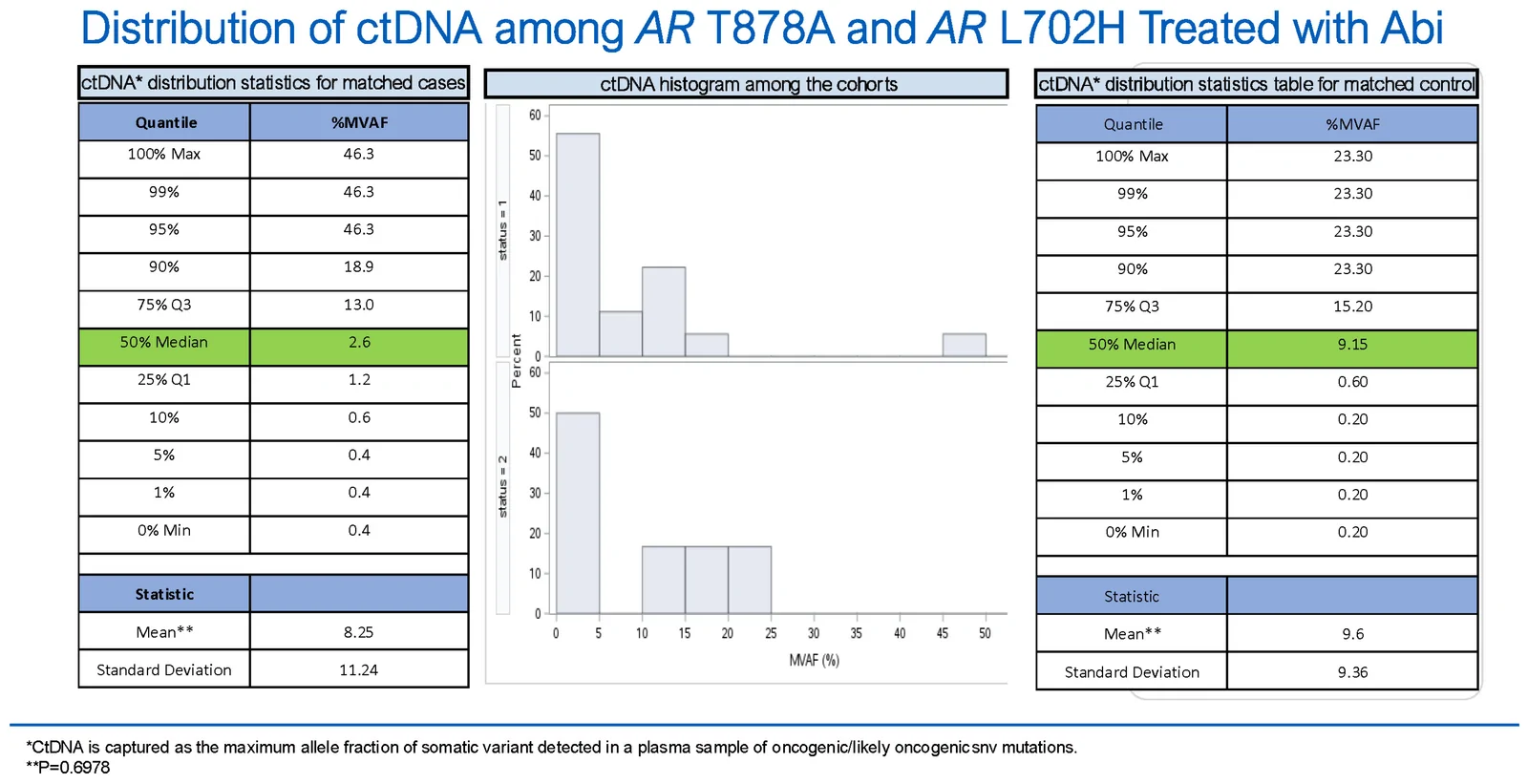
Histogram depicting the distribution of the maximum variant allele fraction detected by cell free DNA among patients in Cohort C with *AR* T878A vs. *AR* L702H treated with Abiraterone.

In total, 36 patients were included in Cohort C, which assessed how outcomes to abiraterone therapy among patients with *AR* T878A alterations compared to outcomes among patients with *AR* L702H alterations. All outcomes are presented as *AR* T878A vs. L702H. The median TTD was 7.1 mo (95% CI 5–9.2 mo) vs. 4 mo (95% CI 3–7.2 mo) (log-rank *p* = 0.3129, aHR 1.69 95% CI 0.83–3.44 *p* = 0.1504) (Figure 3). The median OS was 26.2 mo (95% CI 13.4 mo-not reached) vs. 10.9 mo (95% CI 6.5–16.4 mo) (log-rank *p* = 0.0138, aHR 3.2 95% CI 1.21–8.5, *p* = 0.0196). The median TTNT was 8.3 mo (95% CI 5.7–15.6 mo) vs. 8 mo (95% CI 4 mo-not reached) (log-rank *p* = 0.7068, aHR 1.29 95% CI 0.53–3.13 *p* = 0.5710). The mean MVAF was 8.25% (95% CI 4.48–11.88%) vs. 9.6% (95% CI 5.11–15.21%) (*p* = 0.6978) (Appendix A Figure A5 and Figure A6).

**Figure A7:**
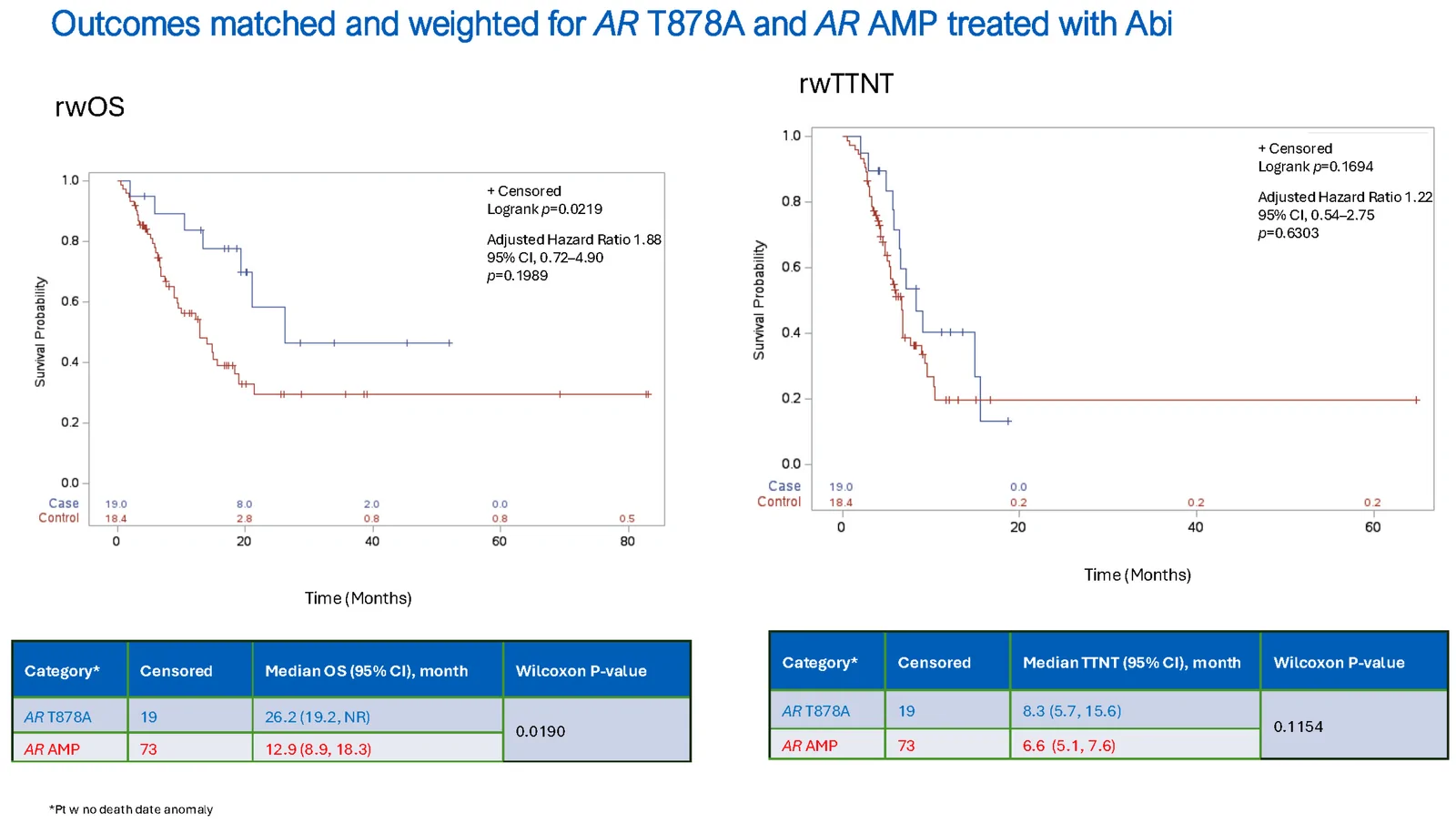
TTNT and OS Cohort D: *AR* T878A vs. *AR* AMP treated with Abiraterone: Left panel depicts KM real-world OS (rwOS). Right panel depicts KM real-world time to next treatment (rwTTNT). Bottom table depicts median OS with 95% confidence intervals and Wilcoxon *p*-value.

**Figure A8:**
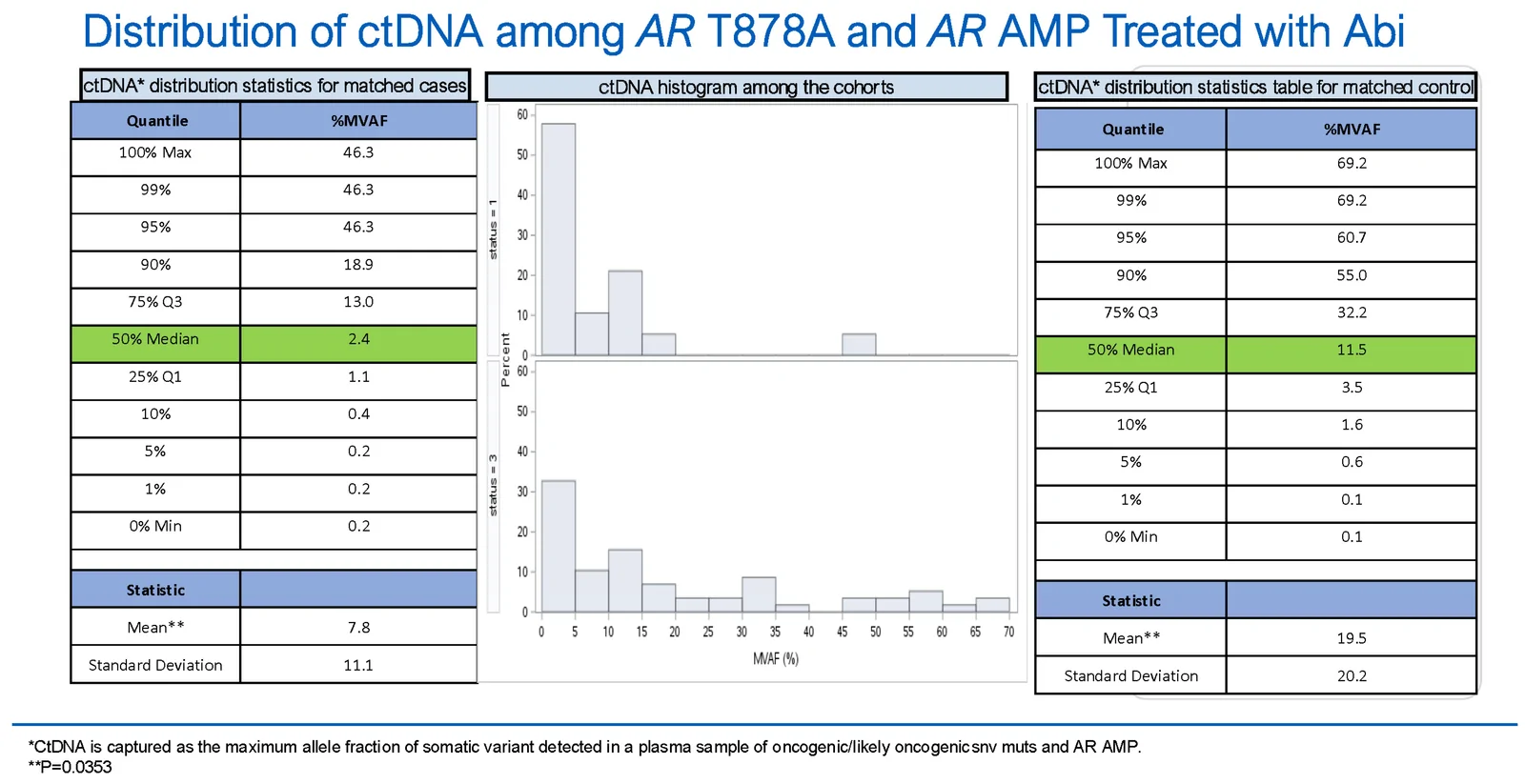
Histogram depicting the distribution of the maximum variant allele fraction detected by cell free DNA among patients in Cohort D with *AR* T878A vs. *AR* AMP treated with Abiraterone.

In total, 38 patients were included in Cohort D, which assessed how outcomes to abiraterone therapy among patients with *AR* T878A alterations compared to outcomes among patients with *AR* amplification. All outcomes are presented as *AR* T878A vs. *AR* Amp. The median TTD was 7.1 mo (95% CI 5–8.5 mo) vs. 4.2 mo (95% CI 3.5–5.4 mo) (log-rank *p* = 0.037, aHR 3.35 95% CI 1.55–7.21 *p* = 0.002) (Figure 3). The median OS was 26.2 mo (95% CI 19.2 mo-not reached) vs. 12.9 mo (95% CI 8.9–18.3 mo) (log-rank *p* = 0.0219, aHR 1.88 95% CI 0.72–4.9, *p* = 0.1989). The median TTNT was 8.3 mo (95% CI 5.7–15.6 mo) vs. 6.6 mo (95% CI 5.1–7.6 mo) (log-rank *p* = 0.1694, aHR 1.22 95% CI 0.54–2.75 *p* = 0.6303). The mean MVAF was 7.8% (95% CI 4.12–11.4%) vs. 19.5% (95% CI 12.83–18.74%) (*p* = 0.0353) (Appendix A Figure A7 and Figure A8).

Across all four cohorts MVAF was not independently associated with TTD, OS or TTNT.

## 3. Discussion

This study is to our knowledge, the largest study to date evaluating real-world outcomes for patients with mAPMR prostate cancer with specific androgen receptor alterations treated with ARPIs. Our findings suggest that patients with *AR* T878A may have superior responses to ARPI therapy relative to patients with *AR* L702H or *AR* amplification as evidenced by the improved real-world time to treatment discontinuation seen in this study particularly with enzalutamide. Our findings are consistent with the distinct pre-clinical profiles of T878A and L702H mutations.

In PSMAfore, a randomized phase 3 trial evaluating lutetium vs. ARPI switch in patients with mAPMR disease who were naïve to taxane-based chemotherapy, the median time to PSA progression in the ARPI change arm was 4.2 months [19]. Based on our analysis, we hypothesize that the response to ARPI could be further optimized by molecularly classifying patients via a cfDNA-based approach in this setting to identify patients with *AR* alterations, such as T878A that likely would yield greater durability. Further prospective studies designed to validate this general management strategy are warranted.

Our study points to the importance of considering the specific *AR* LBD alteration as a potential key determinant of efficacy of AR-targeted therapies. The importance of this may also apply to novel AR-targeted therapeutics in development such as CYP11A1 inhibitors, Proteolysis Targeting Chimeras (PROTACs), and Regulated Induced Proximity TArgeting Chimeras (RIPTACs). While these agents have shown efficacy among patients with *AR* LBD point mutations, that efficacy is not uniform [20,21]. As an example, according to a press release by the manufacturer it has been shown that the *AR* degrader bavdegalutamide is significantly less efficacious in patients with *AR* L702H as compared to other LBD alterations [22]. Further work on the next generation of AR targeting therapies should, as much as possible, stratify results by individual subtypes of AR alterations.

Importantly, while we advocate for further study of individual *AR* point mutations, we acknowledge that our work also highlights the potential challenge of conducting such studies. The overall prevalence of AR LBD point mutations among patients with mAPMR prostate cancer has been estimated to be between 15 and 24% depending on treatment history. Approximately, 60% of these mutations consist of L702H while 33% are T878A. However, a significant fraction of those alterations co-occur with other *AR* LBD point mutations, which can result in changes in the biological and clinical behavior of those patients’ disease. Patients with multiple co-occurring *AR* LBD mutations were omitted from this work, which allowed us to assess outcomes in a more homogenous molecularly defined cohort than prior studies have assessed. However, for this reason this work does not speak to the clinical behavior of many mAPMR prostate cancer patients who harbor multiple *AR* alterations. Further research into the clinical impact of multiple co-occurring *AR* alterations remains an important area for future study. Only 35% of these mutations consist of isolated L702H and only 15% are isolated T878A [6]. Practically, the rarity of individual AR LBD point mutations will make studying the individual behavior of different variants challenging even using large data sets as was done in this study. The relatively small sample sizes, particularly after propensity-score matching seen in this study, reflect the rarity of these specific *AR*-defined molecular subgroups and limit our statistical power and precision. We recommend that nonsignificant comparisons in the present study be considered inconclusive rather than evidence of a trend. Our findings should be viewed as hypothesis generating and require validation in larger cohorts or prospective studies if possible.

Our study has several other notable limitations. Firstly, we note that the claims database this work was based on does not contain detailed clinical information. The average NCI comorbidity index was low relative to other published cohorts of mAPMR prostate cancer patients, suggesting this cohort may have been healthier than typical [23]. PSA levels, history of visceral metastases, and measures of disease burden were not available for review. Therefore, residual confounding from differences in baseline disease severity and underlying disease biology cannot be excluded and may partially contribute to the observed differences in outcomes between cohorts. This possibility is a particularly relevant limitation to the interpretation of our analysis of Cohort B and Cohort D, in which the patients with *AR* Amplified disease had a significantly higher MVAF, which is known to correlate with disease burden and represents a poor prognostic sign [24]. Notably, MVAF was not independently associated with any clinical outcome in our analysis and our multivariable Cox proportional hazard regression analysis, which adjusted for MVAF, continued to show a significant pattern of improved TTD in Cohorts A, B and D.

An additional limitation, again stemming from the limitations in our underlying data is that patients included here may have been treated with a sequence of therapies that may not match clinical practice today. Many patients in this study received ARPI therapy in the 3rd or later line of treatment. The individual lines of treatment used among the patients included in this study were not known and for these reasons the outcomes of the patients examined here may differ in important ways from outcomes seen in modern practice.

Finally, because this work was based on healthcare claims data the radiographic and clinical data needed to document progression free survival and cancer specific survival were not available. We therefore utilized TTD as our primary real-world treatment duration endpoints. Notably reasons for treatment discontinuation were not available in our data set. Prior work has shown that TTD is among the most consistently available outcome metric in real-world data sets and TTD has correlated with PFS in other areas of cancer research [25,26]. While real-world overall survival was also reported in this study, it was not selected as our primary outcome metric because survival data in claims based real-world data is often missing in many patients and because the lack of detailed clinical information as detailed above would limit the interpretability of overall survival in this study [27]. We acknowledge these outcome variables are all real-world surrogate endpoints with limitations and again recommend this study be viewed as hypothesis generating.

## 4. Materials and Methods

### 4.1. Overview

This article is a revised and expanded version of an abstract entitled “Real world outcomes for patients with metastatic castration resistant prostate cancer (mCRPC) and *AR* T878A alterations treated with enzalutamide” which was originally presented at ASCO in Chicago, IL, USA in 2025 [28]. In this analysis, we leveraged the InfinityAI Data Library, which is a real-world clinical-genomic database that links large-scale biopsy profiling with claims-based clinical outcomes from patients with advanced cancers. The database comprises 442,586 patients across more than 50 tumor types, including 34,590 prostate cancer patients. The primary objective of this study was to assess how clinical outcomes to ARPI therapy among patients with mAPMR prostate cancer with an *AR* T878A alteration compared to outcomes among other resistant subtypes. Matched outcomes in this group were compared with those of mAPMR prostate cancer patients treated with enzalutamide or abiraterone who had *AR* L702H alterations and those with *AR* amplification. To be eligible for inclusion in this analysis patients had to be at least 18 years of age, have at least 90 days of follow up available with at least two pharmacy claims post index date and have no death anomaly in claims data. In this study a death anomaly was defined as either a death date that preceded the patient index date or claims based evidence of healthcare utilization occurring more than six months after a recorded death. In addition, patients had to be enzalutamide/abiraterone naïve in the androgen pathway modulation-sensitive (APMS) setting, be treated with enzalutamide/abiraterone in the mAPMR setting and have a pre-ARPI guardant 360 available for analysis. Lastly, given it is known that co-occurring *AR* alteration can have unique biology we limited this analysis to include only patients with a single *AR* alteration.

### 4.2. Matched Clinical Outcomes

For the matched outcomes analyses, patients with *AR* T878A were designated as the case cohort, while patients with *AR* L702H and *AR* Amp were included as control cohorts. Treatment-specific analyses were conducted separately for enzalutamide and abiraterone. For enzalutamide-treatment outcomes, we identified enzalutamide-naïve mAPMR prostate cancer patients as above who initiated enzalutamide in the mAPMR setting; an analogous approach was applied for abiraterone-treated outcomes. Four total matched cohorts were analyzed for patients with the following *AR* alterations: Cohort A consisted of *AR* T878A vs. *AR* L702H treated with enzalutamide, Cohort B consisted of *AR* T878A vs. *AR* Amp treated with enzalutamide, Cohort C consisted of *AR* T878A vs. *AR* L702H treated with abiraterone, and Cohort D consisted of *AR* T878A vs. *AR* Amp treated with abiraterone. Propensity score matching was conducted utilizing a greedy nearest-neighbor algorithm, in which each case was paired with up to four controls, following a 1:4 matching ratio. To minimize order dependence, cases were matched in a random sequence. Matching variables included age (continuous), race, ethnicity, geographic location, baseline NCI Comorbidity Index (continuous), and exact matching on enzalutamide or abiraterone line of therapy.

### 4.3. Statistical Analyses

Chi-squared/Fisher’s exact test was used for the comparison of categorial variables. The Student’s *t*-test was used for continuous variables.

For outcomes analyses overall survival (OS), time to treatment discontinuation (TTD), and time to next treatment (TTNT) among patients with *AR* T878A treated with enzalutamide were compared with the outcomes of patients with *AR* L702H and to those with *AR* Amp using the Kaplan–Meier method. The process was repeated to assess for differences in outcomes among those patients treated with abiraterone. Differences in outcome distributions across cohorts were evaluated using log-rank tests. Multivariable Cox proportional hazards regression analysis was performed for all cohorts using the *AR* T878A group as a reference and incorporated all variables used in propensity score matching (Age, race, ethnicity, geographic region, NCI comorbidity index and line of therapy) as well as maximum variant allele fraction (MVAF). Results are reported as adjusted hazard ratios with 95% confidence intervals. TTD was assessed as the primary outcome of interest. OS and TTNT were assessed as key secondary outcomes. The distribution of ctDNA was compared across cohorts. ctDNA volume was captured as the maximum allele fraction of somatic variants detected in a plasma sample of oncogenic single nucleotide variant mutations. The mean MVAF was compared across cohorts using a Student’s *t*-test. *p* values of <0.05 were considered statistically significant. All analyses were conducted using SAS software package 9.4 (SAS Institute, Cary, NC, USA).

## 5. Conclusions

In conclusion, we present real-world outcomes of patients with mAPMR prostate cancer with *AR* T878A alterations treated with ARPIs. Our findings are hypothesis generating and suggest that patients with *AR* T878A may have a disease that is comparatively more sensitive to ongoing hormonal therapy (i.e., ARPI switch) than other resistant cohorts such as those with *AR* L702H or *AR* amplification.

## Figures and Tables

**Table 1:** Patient demographics, age and NCI comorbidity index are presented as numbers (standard deviations) while other variables are presented as numbers (percent). The first and second columns represent Cohort A (AR T878A/Enzalutamide vs. AR L702H/Enzalutamide). The third and fourth columns represent Cohort B (AR T878A/Enzalutamide vs. AR amplification/Enzalutamide). The fifth and sixth columns represent Cohort C (AR T878A/Abiraterone vs. AR L702H/Abiraterone). The seventh and eighth columns represent Cohort D (AR T878A/Abiraterone vs. AR amplification/abiraterone). LOT = Line of Therapy.

Group (Number)	*AR* T878A/Enza (N = 34)	*AR* L702H/Enza (N = 34)	*AR* T878A/Enza (N = 43)	*AR* AMP/Enza (N = 43)	*AR* T878A/Abi (N = 18)	*AR* L702H/Abi (N = 18)	*AR* T878A/Abi (N = 19)	*AR* AMP/Abi (N = 19)
Age	69 (9.5)	70 (6.6)	69 (9.7)	69 (4.4)	71 (6.0)	72 (4.5)	70 (7.4)	70 (4.1)
NCI Comorbidity Index	0.06 (0.12)	0.05 (0.12)	0.11 (0.23)	0.07 (0.14)	0.05 (0.14)	0.03 (0.08)	0.04 (0.13)	0.02 (0.04)
Race-White	26 (76%)	26 (76%)	33 (77%)	33 (77%)	14 (78%)	15 (83%)	15 (79%)	16 (84%)
Race-Black	4 (12%)	5 (15%)	5 (12%)	4 (9%)	2 (11%)	1 (6%)	2 (11%)	1 (5%)
Race-Other or Unknown	4 (12%)	3 (9%)	5 (11%)	6 (14%)	2 (11%)	2 (11%)	2 (11%)	2 (11%)
LOT-1	5 (15%)	5 (15%)	8 (19%)	8 (19%)	2 (11%)	2 (11%)	2 (11%)	2 (11%)
LOT-2	10 (29%)	10 (29%)	14 (33%)	14 (33%)	3 (17%)	3 (17%)	3 (16%)	3 (16%)
LOT-3	12 (35%)	12 (35%)	12 (28%)	12 (28%)	6 (33%)	6 (33%)	6 (32%)	6 (32%)
LOT-4	3 (9%)	3 (9%)	3 (7%)	3 (7%)	3 (17%)	3 (17%)	3 (16%)	3 (16%)
LOT-5+	4 (12%)	4 (12%)	6 (13%)	6 (13%)	4 (22%)	4 (22%)	5 (26%)	5 (26%)

## Data Availability

This project was based on Guardant Health’s proprietary data set InfinityAI Data Library. The analyses were iterative and involved multiple database queries. The data for the patients treated with enzalutamide were obtained from the database between 17 September 2024 and 2 October 2024. The data for the patients treated with abiraterone were obtained between 16 February 2025 and 3 March 2025. The data are not publicly available. For those interested in reviewing the data please submit a request on the Guardant Health website: https://guardanthealth.com/products/infinityai/ (accessed on 9 March 2022).

## Abbreviations

The following abbreviations are used in this manuscript:

aHR	Adjusted Hazard Ratio
AR	Androgen Receptor
AR AMP	Androgen Receptor Amplification
APMS	Androgen Pathway Modulation-Sensitive
ARPI	AR Pathway Inhibitor
cfDNA	Cell Free Deoxyribonucleic Acid
KM	Kaplan–Meier
LBD	Ligand Binding Domain
LOT	Line of Therapy
PROTAC	Proteolysis Targeting Chimera
NCI	National Cancer Institute
mAPMR	Metastatic Androgen Pathway Modulation-Resistant
mAPMS	Metastatic Androgen Pathway Modulation-Sensitive
MVAF	Maximum Variant Allele Fraction
OS	Overall Survival
PFS	Progression Free Survival
RIPTAC	Regulated Induced Proximity Targeting Chimeras
rwOS	Real World Overall Survival
rwTTD	Real World Time to Treatment Discontinuation
rwTTNT	Real World Time to Next Treatment
TTD	Time to Treatment Discontinuation
TTNT	Time to Next Treatment
